# Prognostic Potential of the Controlling Nutritional Status (CONUT) Score in Predicting All-Cause Mortality and Major Adverse Cardiovascular Events in Patients With Coronary Artery Disease: A Meta-Analysis

**DOI:** 10.3389/fnut.2022.850641

**Published:** 2022-05-09

**Authors:** Godana Arero, Amanuel Godana Arero, Shimels Hussien Mohammed, Ali Vasheghani-Farahani

**Affiliations:** ^1^Department of Public Health, Adama Hospital Medical College, Adama, Ethiopia; ^2^Cardiac Primary Prevention Research Center, Cardiovascular Diseases Research Institute, Tehran University of Medical Sciences, Tehran, Iran; ^3^Universal Scientific Education and Research Network (USERN), Addis Ababa, Ethiopia; ^4^Ethiopian Public Health Institute, Addis Ababa, Ethiopia; ^5^Department of Clinical Cardiac Electrophysiology, Tehran Heart Center, Tehran University of Medical Sciences, Tehran, Iran

**Keywords:** coronary artery disease, mortality, cardiovascular events, meta-analysis, controlling nutritional status score

## Abstract

**Background:**

As defined by the Controlling Nutrition Status (CONUT) score, the prognostic significance of nutritional status has attracted attention in patients with cardiovascular disease. This meta-analysis aimed to determine the importance of CONUT score for prediction of all-cause mortality and major adverse cardiovascular events (MACE) in adult patients with coronary artery disease (CAD).

**Methods:**

Observational studies conducted to evaluate the association of CONUT score with adverse clinical outcomes in patients with CAD were included. We searched MEDLINE, Embase, Scopus, Cochrane library, Google scholar, medRxiv pre-print as well as Science Direct search engine for studies published from the inception of each database until March 21, 2022. Studies reporting the utility of CONUT score in prediction of all-cause mortality and MACE among patients with CAD were eligible. Predictive potential of the CONUT score were summarized by pooling the multivariable adjusted hazard ratio (aHR) with 95% CI for the malnourished vs. normal nutritional status or per point CONUT score increase.

**Results:**

Of 2,547 screened citation, nine observational studies involving 81,257 patients with CAD were analyzed. Malnutrition defined by the CONUT score was associated with significantly increased risk of all-cause mortality when compared with the normal nutritional state (aHR for mild, moderate, and severe malnutrition, respectively: (1.21 [95% CI: 1.15–1.27], *I*^2^ = 0%), (1.53 [95% CI: 1.26–1.84], *I*^2^ = 84%), and (2.24 [95% CI: 1.57–3.19], *I*^2^ = 77%). Similarly, moderate (aHR 1.71 [95% CI: 1.44–2.03], *I*^2^ = 0%) and severe (aHR 2.66 [95% CI: 1.82–3.89], *I*^2^ = 0%) malnutrition was associated with a significantly higher risk of MACE compared with the normal nutritional state. Additionally, per point increase in the CONUT score was correlated with 20 and 23% additional risk of all-cause mortality and MACE, respectively.

**Conclusion:**

As defined by the CONUT score, malnutrition is an independent predictor of all-cause mortality and MACE in CAD patients. Nutritional assessment with CONUT score could allow clinicians to identify patients with CAD at high risk for adverse clinical outcomes.

## Introduction

Coronary artery disease (CAD) remains the leading single cause of death worldwide (1, 2). Since the dramatic development of pharmacological and interventional therapies, the prognosis of CAD patients has improved. However, mortality in CAD patients remains high, and CAD patients are at risk for developing major adverse cardiovascular events (MACE) (3, 4). Therefore, it is particularly important to choose a reasonable preventive strategy for CAD and identify the risk factors leading to poor outcomes.

Malnutrition is known to be associated with poor clinical outcomes in a variety of diseases (5–8). In patients with cardiovascular diseases, evidence indicates that malnutrition is associated with increased in-hospital mortality, mid-to-long-term mortality, and cardiovascular events (9–12). Although nutritional assessment could be a useful predictor of clinical outcome in patients with cardiovascular disease, the lack of a unified definition and gold standard methods for evaluating nutritional status makes it difficult to diagnose malnutrition. Albumin, body weight, body mass index, cholesterol, and other indices are often used to measure nutritional status, although they are often inaccurate (13). Several tools for assessing nutritional status have been developed, including the Geriatric Nutritional Risk Index (GNRI), Prognostic Nutritional Index (PNI), Controlling Nutritional Status (CONUT) score, and Mini Nutritional Assessment (MNA). These nutritional assessment tools showed prognostic value in patients with malignancy, CAD, heart failure and, peripheral arterial diseases (8, 9, 11, 14, 15).

The controlling Nutritional Status (CONUT) score has been reported to be a simple and efficient screening tool for evaluating malnutrition status, especially for early detection and ongoing monitoring of nutritional status in hospitalized patients (16). Current studies suggest that CONUT is associated with short- and long-term prognoses in some diseases (9, 14, 15, 17). The CONUT score is calculated based on the parameters of serum albumin, total cholesterol and, total lymphocyte levels, consisting of an immune-nutritional index reflecting protein/lipid metabolism and immunocompetence (16). According to the CONUT score, individuals with a CONUT score of 0–1 have a normal nutritional status, those with a CONUT score of 2–4 have a mild a moderate degree of malnutrition, and those with a CONUT score of 9–12 have a severe degree of malnutrition (16). The lower level of the laboratory variables used to calculate the CONUT score has shown to be associated with disease progression and mortality in patients with CAD (18–23). Furthermore, when compared to other nutrition-related measures in patients with CAD, recent studies have demonstrated that the CONUT score has the highest predictive ability for mortality and MACE (24, 25). However, no previous meta-analysis has specifically addressed the predictive value of CONUT score for all-cause mortality and MACE in this population group. Therefore, we sought to conduct a meta-analysis to assess the association of malnutrition defined by the CONUT score with all-cause mortality and MACEs in patients with CAD.

## Methods

### Search Strategy and Study Selection

In reporting the present study, we followed the recommendation of Meta-analysis of Observational Studies in Epidemiology guideline (MOOSE) (26) and the preferred Reporting Items for Systematic Reviews and Meta-Analyses 2020 (PRISMA) 2020 statement (27). We conducted the literature search by screening MEDLINE, Embase, Scopus, Cochrane library, Google scholar, medRxiv pre-print as well as Science Direct search engine. Hand search was done in publishers and journals websites databases in March 2022 for studies published from the inception of each database until March 21, 2022. The following keywords in combination were applied for literature search: “Controlling nutritional status” OR “CONUT” OR “Malnutrition” AND “coronary artery disease” OR “coronary heart disease” OR “acute coronary syndrome” OR “myocardial infarction” OR “unstable angina pectoris” (Supplementary Table 1). The search was run a second time before finalizing the retrieved articles and incorporating any additional identified studies. In addition, we checked the reference list of related articles to identify potentially missing studies. Only articles published in English were considered.

We included observational studies conducted to assess the association of CONUT score with all-cause mortality or MACEs in adult patients with coronary artery disease. No restriction was implemented regarding the sex of the patients. The exclusion criteria included studies that lack a detailed risk summary for the outcomes or reporting unadjusted risk estimates. According to the inclusion and exclusion criteria, GA and AGA independently checked titles and abstracts of search results retrieved from the databases. The same researchers independently reviewed full text articles. The same researchers independently reviewed full text articles. All reviewed and excluded articles were documented on an excel spreadsheet with annotations for reasons of exclusion. In case of discrepancies, they were resolved by inter-researcher discussion with SHM and AVF.

### Data Extraction and Assessment of Quality of the Included Studies

GA and AGA extracted data from the final set of included studies for study characteristics, baseline population characteristics, and results in a standardized evidence table. SHM checked these data for accuracy. Disagreements were managed through discussion between researchers. We assessed the methodological quality of the included studies using the Quality in Prognostic Factor Studies (QUIPS) checklist (28). The ratings for individual study were compared between the two researchers and differences were resolved by consensus.

### Statistical Analysis

The main analysis was to determine the predictive value of baseline CONUT on all-cause mortality and MACE. The hazard ratio (HR) was used to summarize the estimates representing the risk of all-cause mortality and MACE. The *I*^2^ -statistic and the Cochrane Q test were used to assesses the heterogeneity between studies, with statistical significance set at *I*^2^ ≥ 50% or *p* < 0.10. We used a random effects model and the generic inverse variance method to calculate the summary of HR in case of significant heterogeneity. Otherwise, a fixed-effect model was selected. Each study was excluded from the meta-analysis one at a time, and a sensitivity analysis was performed to examine the effect of each study by assessing the degree of change in the magnitude and significance of the effects of exposure. We used CMA 3 and RevMan 5.4. A 2-sided *P*-value at 5% level is considered significant.

## Results

### Study Selection

**FIGURE 1:**
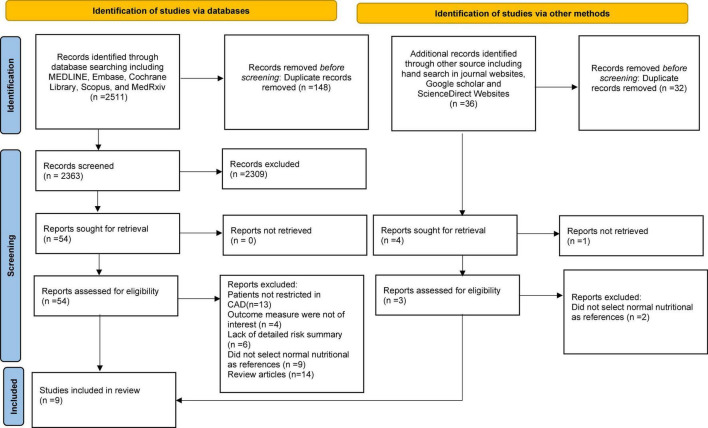
Flow chart showing studies selection process.

The literature search and selection process are depicted in the PRISMA flow diagram (Figure 1). A total of 2,547 references were retrieved from the database using the described methodology. Of these, 184 duplicate publications and 2,311 references were excluded after scanning the titles and abstracts. The full texts of 57 references were evaluated. After a more detailed review,48 references were excluded for different reasons. For the final analysis, nine (13, 24, 25, 29–34) observational studies were included.

### Study Characteristics

The relevant characteristics of the studies included in the meta-analysis are in detailed in Table 1. The included studies were published between 2016 and 2022 and were conducted in Italy, China, Turkey, Taiwan, Japan, and Spain. Seven were retrospective studies (13, 25, 30–34) and two were prospective (24, 29). The number of patients in the eligible studies ranged from 253 to 46,485, for a total of 81,257 CAD patients. The mean age of the participants was 68.6 years old and 71% were male. The mean follow-up period was 4.37 years. The quality assessment of reviewed studies is presented in Supplementary Table 2. The assessment suggested a low-moderate risk of bias for each item. The most common reason that studies have a moderate risk of bias is an inadequate adjustment for confounding factors.

**TABLE 1:** Main characteristic of the included studies.

References	Country	Study design	Age, years	Patients (%Men)	Cut-off value of CONUT	Outcomes	Follow-up, years	Adjusted variables
Basta et al. (29)	Italy	Prospective	65.7	STEMI 945 (75)	Per point increase	All-cause mortality	2	Age and gender
Chen et al. (13)	Taiwan	Retrospective	71.5	CAD 3,118 (81.5)	Per point increase. Mild risk (2–4). Moderate risk (5–8). Severe risk (9–12)	MACE	4.8	Age, gender, BMI, DM, HTN, statins, LDL, and HDL
Chen et al. (32)	China	Retrospective	69.5	CAD 21,479 (72.6)	Per point increase. Mild risk (2–4). Moderate risk (5–8). Severe risk (9–12)	All-cause mortality	5.16	Age, gender, AF, DM, eGFR < 60, CKD, anemia, CHF, ACE/ARB, BBs, statins
Kalyoncuoğlu et al. (24)	Turkey	Prospective	68.5	NSTEMI 253 (75.5)	Per point increase	MACE	1.7	Age, BMI, DM, eGFR, LVEF, and PNI
Kunimura et al. (30)	Japan	Retrospective	68.4	CAD 1,004 (67.4)	Per point increase	MACE	4.8	Age, gender, and current smoker, DM, HTN, dyslipidemia, eGFR, EF, BNP, and previous history of PCI or CABG.
Liu et al. (34)	China	Retrospective	63.1	CAD 46,465 (75.8)	Mild risk (2–4). Moderate risk (5–8). Severe risk (9–12)	All-cause mortality	5.1	Age, sex, PCI, HTN, AF, DM, CKD, anemia, CHF, ACE/ARB, BBs, and statin.
Raposeiras-Roubín et al. (12)	United States	Retrospective	66.2	ACS 5,062 (74.5)	Per point increase. Mild risk (2–4). Moderate risk (5–8). Severe risk (9–12)	All-cause mortality MACE	3.6	Age, sex, BMI, HTN, dyslipidemia, DM, prior MI, CHF, PAD, COPD, prior cancer, AF, type of ACS, Killip class > II, creatinine (mg/dl), LVEF, multivessel coronary artery disease, PCI, complete revascularization, therapy at discharge (dual antiplatelet therapy, BBs, ACE/ARB, and statins), and GRACE risk score.
Wada et al. (31)	Japan	Retrospective	66.4	CAD 1,987 (82.8)	Per point increase	MACE	7.4	Age, gender, BMI, CKD, current smoker, DM, dyslipidemia, HTN, LVEF, multivessel disease,and statins
Yıldırım et al. (33)	Turkey	Retrospective	73.1	NSTEMI 915 (51.6)	Per point increase	All-cause mortality	5.4	BMI, PNI, and GNRI.

*BMI, Body mass index; DM, Diabetes mellitus; HTN, Hypertension; LDL; HDL; AF, Atrial fibrillation; eGFR; CKD, chronic kidney disease; CHF, congestive heart failure; ACE/ARB, angiotensin converting enzyme inhibitors or angiotensin receptor blockers; BBs, beta-blockers; LVEF, left ventricular ejection fraction; PNI, prognostic nutritional index; EF, Ejection fraction; BNP, brain natriuretic peptide; coronary artery bypass grafting, CABG; PCI, percutaneous coronary intervention; MI, Myocardial infarction; PAD, peripheral artery disease; COPD, chronic obstructive pulmonary disease; ACS, Acute coronary syndrome; GRACE, Global Registry of Acute Coronary Events; GNRI, geriatric nutritional risk index; STEMI; ST-elevation myocardial infarction, NSTEMI; non- ST-elevation myocardial infarction; CAD, Coronary artery disease.*

### Categorical Analysis of Controlling Nutrition Status Score on All-Cause Mortality and Major Adverse Cardiovascular Events

Four studies (13, 25, 32, 34) provided the categorical analysis of the CONUT score. As shown in Table 2, the meta-analysis indicated malnutrition defined by the CONUT score was significantly associated with higher risk for all-cause mortality when compared with the normal nutritional state (adjusted hazard ratio (aHR) for mild, moderate, and severe degrees of malnutrition, respectively: [1.20 (95% CI: 1.14–1.26), *I*^2^ = 0%], [1.53 (95% CI: 1.26–1.84), *I*^2^ = 84%], and [2.24 (95% CI: 1.57–3.19), *I*^2^ = 77%]. Similarly, moderate [aHR 1.71 (95% CI: 1.44–2.03), *I*^2^ = 0%] and severe [aHR 2.66 (95% CI: 1.82–3.890, *I*^2^ = 0%] degrees of malnutrition were significantly increased risk of MACE when compared with the normal nutritional status. Statistically, a non-significant association was observed for mild [aHR 1.05 (95% CI: 0.92–1.20), *I*^2^ = 0%] degree of malnutrition when compared with the normal nutritional state for MACE. There was no evidence of heterogeneity for the meta-analysis of the association of categorical CONUT score and MACE.

**TABLE 2:** Meta-analysis on all-cause mortality and MACE by categorical analysis of CONUT score.

Outcomes (No. of study)	CONUT, categorical (normal nutrition as reference)	HR [95% CI]	*I*^2^,%	*P*-value
**All-cause mortality (3)**				
	Mild risk	1.20 [95% CI: 1.14–1.26]	0	<0.00001
	Moderate risk	1.53 [95% CI: 1.26–1.84]	84	<0.0001
	Severe risk	2.24 [95% CI: 1.57–3.19]	77	<0.00001
**MACE (2)**				
	Mild risk	1.05 [95% CI: 0.92–1.20]	0	0.48
	Moderate risk	1.71 [95% CI: 1.44–2.03]	0	<0.00001
	Severe risk	2.66 [95% CI: 1.82–3.89]	0	<0.00001

### Continuous Analysis of Controlling Nutrition Status Score on All-Cause Mortality and Major Adverse Cardiovascular Events

**FIGURE 2:**
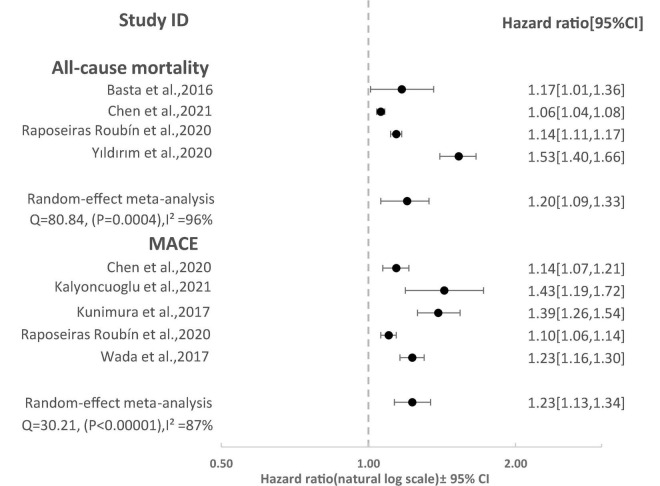
Forest Plot showing pooled hazard ratio with 95% CI of all-cause mortality and major adverse cardiovascular events (MACE) for per point increase in CONUT score.

**FIGURE 3:**
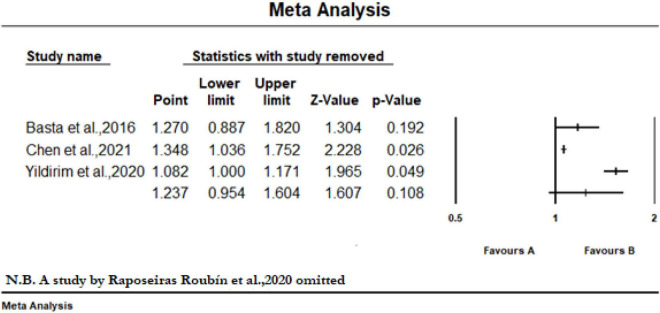
Sensitivity analysis of included studies to determine the pooled hazard ratio with 95% CI of all-cause mortality for per point increase in CONUT score when a study by Raposeiras Roubín et al. (25) was omitted.

Eight studies reported the predictive value of the CONUT scores by continuous analysis. As shown in Figure 2, a random-effect meta-analysis indicated that the pooled aHR of all-cause mortality and MACE was (1.20 [95% CI: 1.09–1.33], *I*^2^ = 96%) and (1.23 [95% CI: 1.13–1.34] *I*^2^ = 87%), respectively, for an increase CONUT score per point, with high heterogeneity. Sensitivity analyses showed that the overall effects remained statistically significant when the individual studies were omitted from the effect size calculation for MACEs. However, for all-cause mortality, the effect reached non-significance in one case when a study by Raposeiras Roubín et al. (25) was omitted (Figure 3).

### Publication Bias

We did not construct the funnel plots or preformed the Begg’s test and Egger’s test to assess the publication bias due to the less than recommended arbitrary minimum number of studies (35).

## Discussion

Our meta-analysis showed that malnutrition, as defined by the CONUT, was associated with higher risk of all-cause mortality and MACE in CAD patients. CAD patients with a mild, moderate, and severe degree of malnutrition had a 1. 25-, 1. 62-, and 2.49-fold exaggerated risk of all-cause mortality, respectively, when compared with the normal nutritional state. Likewise, CAD patients with a moderate and severe degree of malnutrition had 1.71- and 2.66-fold increased risk of MACEs, respectively. Moreover, per point increase in CONUT score was associated with 20 and 23% higher risk of all-cause mortality and MACE, respectively. The current meta-analysis suggests that malnutrition can affect the trajectory of CAD prognosis. These findings also confirm the availability of evidence of nutritional assessment by CONUT score in risk stratification of CAD patients.

Malnutrition is common among patients with CAD. Its prevalence varies with various malnutrition indices. Raposeiras Roubín et al. (25) reported that the prevalence of undernutrition in patients with the acute coronary syndrome (ACS) ranged from 9% for the Prognostic Nutritional Index, 50% for the CONUT, and up to 60% for the Nutritional Risk Index. Similarly, Tonet et al. (36) showed that the percentage of ACS patients with malnutrition was 44% using the Mini Nutritional Assessment (MNA). Another recent study conducted to assess the magnitude of malnutrition in diabetic patients with CAD using the CONUT score demonstrated that 60.5% of patients with both CAD and diabetes suffered from malnutrition (37). Moreover, in concordant with our findings, malnutrition determined by the various malnutrition indices was significantly associated with adverse clinical outcomes in patients with CAD (11, 38–40). Therefore, clinicians need to integrate malnutrition detection into their daily practice.

The exact mechanisms of predictive values of CONUT score in CAD remain unclear. CONUT is a simple and efficient screening tool that reflects the nutritional status and immune function of the body (41). The CONUT is based on total lymphocyte count, total cholesterol, and serum albumin (16). The lower level of these laboratory variables has shown to be associated with disease progression and mortality in patients with CAD (18–23). The synthesis of albumin is affected by both nutritional intake and systemic inflammation (19). Several studies have reported that the link between low serum albumin levels and adverse clinical outcomes in patients with heart failure and inflammation has been suggested as a major etiology of low albumin levels (42). Therefore, serum of albumin may indicate both systemic inflammation and nutritional status. Inflammation plays a crucial role in the initiation and progression of atherosclerosis and its acute clinical manifestations (43). Moreover, hypoalbuminemia may indicate persistent injury to the arteries and the progression of atherosclerosis and thrombosis (19). Disruption of cholesterol homeostasis, which occurs as part of the innate immune response, can exacerbate the inflammatory response that causes atherosclerosis (44, 45). The correlation between elevated serum cholesterol levels and cardiovascular disease is well established (46). However, the predictive value of total cholesterol is surprising due to the inverse relationship with adverse clinical outcomes (47). These relationships corroborate the idea of reverse epidemiology, wherein low level of conventional risk factors can seem deleterious (48, 49). Though the cause of the lipid paradox remains unclear, beside other possible explanations, cholesterol-lowering effect of systemic inflammation and malnutrition has been strongly suggested (13, 50, 51). Furthermore, patients with low cholesterol may not receive aggressive statin treatment, and relatively low statin use perhaps may be responsible for high risk within the high-COUNT score group. Malnutrition has been introduced as the most prevalent cause of immunodeficiency (52). Lymphocyte count reduction was shown to be independently and significantly associated with adverse clinical outcomes in CAD (22, 23). Low total lymphocyte level was also associated with higher mortality and was a predictor of prognosis (53, 54). These three laboratory variables contribute to the pathophysiology of CAD (55). Considering the above-mentioned pieces of evidence, the CONUT score may be a reasonable tool for evaluating the nutritional status of the CAD population. Previously published studies have shown that the CONUT score predicts adverse clinical outcome in a population with heart failure (9), stroke (56, 57), hypertension (41), and cancers (58–60).

Despite the increasing number of studies showing the risk of malnutrition, malnutrition is often not listed as a comorbidity of CAD. Our findings strongly support the need for clinicians to practice early identification of malnutrition in high-risk populations. This improves risk stratification and guides subsequent secondary preventive interventions. The variables required to calculate the CONUT score are widely available in routine laboratory tests, malnutrition can be systematically screened in CAD settings regardless of the stage (acute or stable). Malnutrition is a modifiable risk factor. CAD patients with malnutrition should be given meaningful and effective nutrition guidance. However, clinical studies have not shown whether nutritional interventions can improve the prognosis of CAD patients with malnutrition.

Our study should be viewed in the context of certain important limitations. First, we combined only observational studies. All limitations of observations must be taken into account. Second, total cholesterol—a specific variable of the CONUT—might be affected by the use of statins therapy, which could have confounded the assessment of nutritional status using the CONUT score. Third, low to high-degree heterogeneity was observed in the included studies. Different subtypes of patients with CAD, definitions of MACEs, or intervals of follow-up duration may be correlated to the observed heterogeneity. Therefore, the analysis should be interpreted cautiously. Fourth, none of the included studies were able to provide information on preventive medication and adherence to medications given during follow-up. Therefore, we were unable to evaluate the effectiveness of prophylaxis in preventing adverse clinical outcomes. Finally, although we intended to assess potential publication bias and perform a subgroup analysis based on CAD subtype (i.e., ACS and chronic CAD) combined in the meta-analysis, it was not possible due to the less than recommended arbitrary minimum number of studies which under power any of these methods.

## Conclusion

As defined by the Controlling Nutrition Status (CONUT) score, malnutrition is an independent predictor of all-cause mortality and MACE in CAD patients. Nutritional assessment with CONUT score could allow clinicians to identify patients with CAD at high risk for adverse clinical outcomes.

## Data Availability Statement

The original contributions presented in the study are included in the article/Supplementary Material, further inquiries can be directed to the corresponding author/s.

## Author Contributions

GA, AV-F, AA, and SM: study conception, design, and revising the article critically for important intellectual content. GA and AA: acquisition of data, analysis, and interpretation of data, and statistical analysis. GA: drafting the article. All authors read and approved the final version of the study to be published.

## Conflict of Interest

The authors declare that the research was conducted in the absence of any commercial or financial relationships that could be construed as a potential conflict of interest.

## Publisher’s Note

All claims expressed in this article are solely those of the authors and do not necessarily represent those of their affiliated organizations, or those of the publisher, the editors and the reviewers. Any product that may be evaluated in this article, or claim that may be made by its manufacturer, is not guaranteed or endorsed by the publisher.
